# Genes Belonging to the Insulin and Ecdysone Signaling Pathways Can Contribute to Developmental Time, Lifespan and Abdominal Size Variation in *Drosophila americana*

**DOI:** 10.1371/journal.pone.0086690

**Published:** 2014-01-28

**Authors:** Micael Reis, Inês Páscoa, Helder Rocha, Bruno Aguiar, Cristina P. Vieira, Jorge Vieira

**Affiliations:** IBMC – Instituto de Biologia Molecular e Celular, University of Porto, Porto, Portugal; University of Lausanne, Switzerland

## Abstract

Even within a single genus, such as *Drosophila*, cases of lineage-specific adaptive evolution have been found. Therefore, the molecular basis of phenotypic variation must be addressed in more than one species group, in order to infer general patterns. In this work, we used *D. americana*, a species distantly-related to *D. melanogaster,* to perform an F2 association study for developmental time (DT), chill-coma recovery time (CRT), abdominal size (AS) and lifespan (LS) involving the two strains (H5 and W11) whose genomes have been previously sequenced. Significant associations were found between the 43 large indel markers developed here and DT, AS and LS but not with CRT. Significant correlations are also found between DT and LS, and between AS and LS, that might be explained by variation at genes belonging to the insulin and ecdysone signaling pathways. Since, in this F2 association study a single marker, located close to the *Ecdysone receptor* (*EcR*) gene, explained as much as 32.6% of the total variation in DT, we performed a second F2 association study, to determine whether large differences in DT are always due to variation in this genome region. No overlapping signal was observed between the two F2 association studies. Overall, these results illustrate that, in *D. americana,* pleiotropic genes involved in the highly-conserved insulin and ecdysone signaling pathways are likely responsible for variation observed in ecologically relevant phenotypic traits, although other genes are also involved.

## Introduction

The complete description of the molecular basis of variation for quantitative traits in natural populations of different species is essential to understand how genetic variation for adaptive traits is maintained, in general, and whether the adaptive phenotypic variation observed within and between species is caused by the same variable loci [1]. In *Drosophila*, most of the work regarding this issue has been done using species from the *melanogaster* group (see for instance, [2]–[8]). However, other groups of species must be studied as well since, even within a single genus such as *Drosophila*, cases of lineage-specific adaptive evolution have been found (see for instance, [9]–[13]). Moreover, genes that have been reported as harboring variability that explains within species phenotypic variation in *D. melanogaster* have been found to be missing in distantly related *Drosophila* species (see for instance, [14]).

*Drosophila americana* (*virilis* group) is becoming an important model for comparative studies. This species is distantly related to *D. melanogaster* since the two lineages have been diverging for about 40 million years [15]. The large effective population size and the geographical distribution along the USA imply a large amount of nucleotide and phenotypic variation. Since *D. americana* is easy to collect across the entire range of the distribution and it is easy to maintain in the laboratory, a large number of strains are already available. Little population structure was observed for this species besides that caused by chromosomal inversions and a chromosomal fusion, for which molecular markers are available. The genome of strains H5 and W11 has been sequenced and assembled (for a detailed discussion see Fonseca et al. [16]).

Here, we present the results of one F2 association study involving the two *D. americana* strains whose genome has been sequenced (H5 and W11; [16]), and four phenotypic traits that are likely ecologically important (developmental time (DT), chill-coma recovery time (CRT), abdominal size (AS) and lifespan (LS)). Significant correlations have been found between DT and LS and AS and LS that can be explained by variation at genes belonging to the insulin and the ecdysone signaling pathways. Since, in this F2 association study a single marker, located close to the *Ecdysone receptor* (*EcR*) gene, explained as much as 32.6% of the total variation in DT, we performed a second F2 association study where the slow developing strain W11 was replaced by another slow developing strain (W37), to determine whether large differences in DT are always due to variation in this genome region. No overlapping signal was observed between the two F2 association studies. Overall, these results illustrate the likely role of pleiotropic genes involved in the insulin and ecdysone pathways, but also of other genes, in the setting of likely ecologically relevant phenotypic traits.

## Materials and Methods

### Marker Development

A set of 43 large indel markers distributed along the five major chromosomes of *D. americana* was developed based on the available genome sequence for strains H5 and W11 [16]. The markers locations, as well as the variants that are segregating in the strains (H5, W11 and W37) used in the F2 association studies (H5♂xW11♀ and H5♂xW37♀) are shown in Fig. S1. Primers and PCR amplification conditions used in this work are shown in Table S1. All PCR products were visualized on a UV transilluminator after electrophoresis using SGTB buffer (Grisp, Portugal) in 2% agarose gels stained with ethidium bromide.

### F2 association Studies

The three isofemale strains used in the F2 association experiments were established with flies collected at the end of July and beginning of August 2004 at Lake Wappapelo, Missouri (W11 and W37) and at Lake Hurricane, Mississippi (H5) and have been kept in the lab at room temperature (20–22°C) in large vials with about 20–50 individuals. Then, these strains were transferred to a controlled temperature chamber at 25°C, with 12/12 hours of light/dark cycles, one generation before starting the experiment. The F2 association experiments have been performed under the latter conditions. The *D. americana* F2 association study involving strains H5 and W11 is described in detail by Reis et al. [14]. Briefly, 131 F2 *D. americana* males showing extreme phenotypes, after excluding all individuals that show phenotypic values for any of the four traits (DT, CRT, AS and LS) in the second third of the distribution (intermediate values), were selected out of 975 individuals. The first trait to be measured was DT. For this purpose, each of the 83 second generation crosses (F1) were transferred to new flasks every day in order to obtain the precise period of time, in days, between oviposition and adult eclosion. The resulting F2 males were then individually collected. When F2 males were 10 days old (young adult flies), individual CRT was measured, in seconds, at 25°C after four hours of cold exposure at 0°C. Flies must be able to stand up on their legs in order to be considered completely recovered. Individual photographs were taken when individuals were 20 days old, using a stereomicroscope Nikon ZMS 1500 H. The resulting JPG files were saved with a resolution of 1600×1200 pixels. Relative AS was estimated by counting the number of pixels in the picture that correspond to this structure, using Adobe Photoshop H (Adobe, USA). The flies were then transferred to new vials and kept until they died, in order to measure LS, in days.

For the F2 association study involving the H5 and W37 strains, 130 individuals showing 14 or less days and 18 or more days of DT (time between oviposition and adult eclosion) were selected. These three strains (H5, W11 and W37) were selected, since they showed remarkable differences regarding the studied phenotypic traits and they show the same chromosomal rearrangements.

For both association studies only males were used, in order to avoid a possible sex effect that might exist. Genomic DNA was then extracted using the QIAamp DNA Mini Kit from QIAGEN (Izasa Portugal, Lda.) according to the manufacturer’s instructions, and the individuals were genotyped using the 43 indel markers described above.

Genotype – phenotype associations were tested using non-parametric tests as implemented in SPSS Statistics 19.0 (SPSS Inc., Chicago, Illinois) and significant values were corrected using the sequential Bonferroni correction for multiple testing. Using the same software, non-linear as well as linear regression analyses (including a constant) were performed between the phenotype observed for each genotyped individual and the expected phenotype (average of the phenotype obtained for each genotypic class), in order to estimate the amount of phenotypic variation explained by variation in the indel markers.

### Candidate Genes

Gene Ontology was used as a proxy to identify *D. melanogaster* putative candidate genes for the four phenotypic traits measured in this study. Then, using FlyBase (http://flybase.org), we identified the *D. virilis* orthologous genes, since this species is the most closely-related to *D. americana* (approximately 4.1My of independent divergence [17]) with an annotated genome, and for which information on orthologous genes is available. In the few cases where orthology information is unavailable, putative orthologous genes were identified using BLASTP search and the *D. melanogaster* protein sequences as query.

The general GO terms selected were GO: 0007476 (imaginal disc-derived wing morphogenesis) for DT, GO: 0008340 (determination of adult lifespan) for LS, GO: 0040014 (regulation of multicellular organism growth) for AS and GO: 0009409 (response to cold) for CRT.

There are 318 genes included in the GO term: imaginal disc-derived wing morphogenesis, along the major *D. melanogaster* chromosomes, but eight of these genes (*amn*; *CG1678*; *CG12717*; *salm*; *CG11226*; *SF1*; *smp-30*; *tal-1A*) have no gene correspondence in *D. virilis*. Moreover, three orthologous genes (*GJ18461*/*Stim*; *GJ22504*/*Pka-C3*; *GJ22515*/*crc*) in *D. virilis* are present in small scaffolds not assigned to any of the Muller’s elements making it hard to determine their cytological location. After excluding these genes there are 307 putative candidate genes for DT differences in *D. americana.*

A total of 147 genes are associated with the GO term: determination of adult life span, but eight of these genes (*CG11700*; *mthl3*; *Acp62F*; *mthl2*; *mthl6*; *mthl7*; *Indy-2*; *mthl12*) have no gene correspondence in *D. virilis*. *bmm* and *fwd* genes show two homolog sequences each, in *D. virilis* and were included in the list of putative candidate genes. Moreover, two orthologous genes (*GJ15405*/*Cdk5alpha*; *GJ14992*/*chico*) in *D. virilis* are present in small scaffolds not assigned to any of the Muller’s elements. After excluding/introducing these genes there are 139 putative candidate genes for LS differences in *D. americana.*

The GO term: regulation of multicellular organism growth comprises 41 genes but one ortholog (*GJ14992*/*chico*) in *D. virilis* is present in a small scaffold not assigned to any of the Muller’s elements. After excluding this gene there are 40 putative candidate genes for AS differences in *D. americana*).

Finally, for CRT there are 11 genes associated with the GO term: response to cold but three of these genes (*brv-3*; *brv-2*; *smp-30*) have no correspondence with any *D. virilis* gene and therefore, only eight putative candidate genes could explain CRT differences in *D. americana*.

## Results

### H5♂xW11♀ F2 Association Study

**Figure 1:**
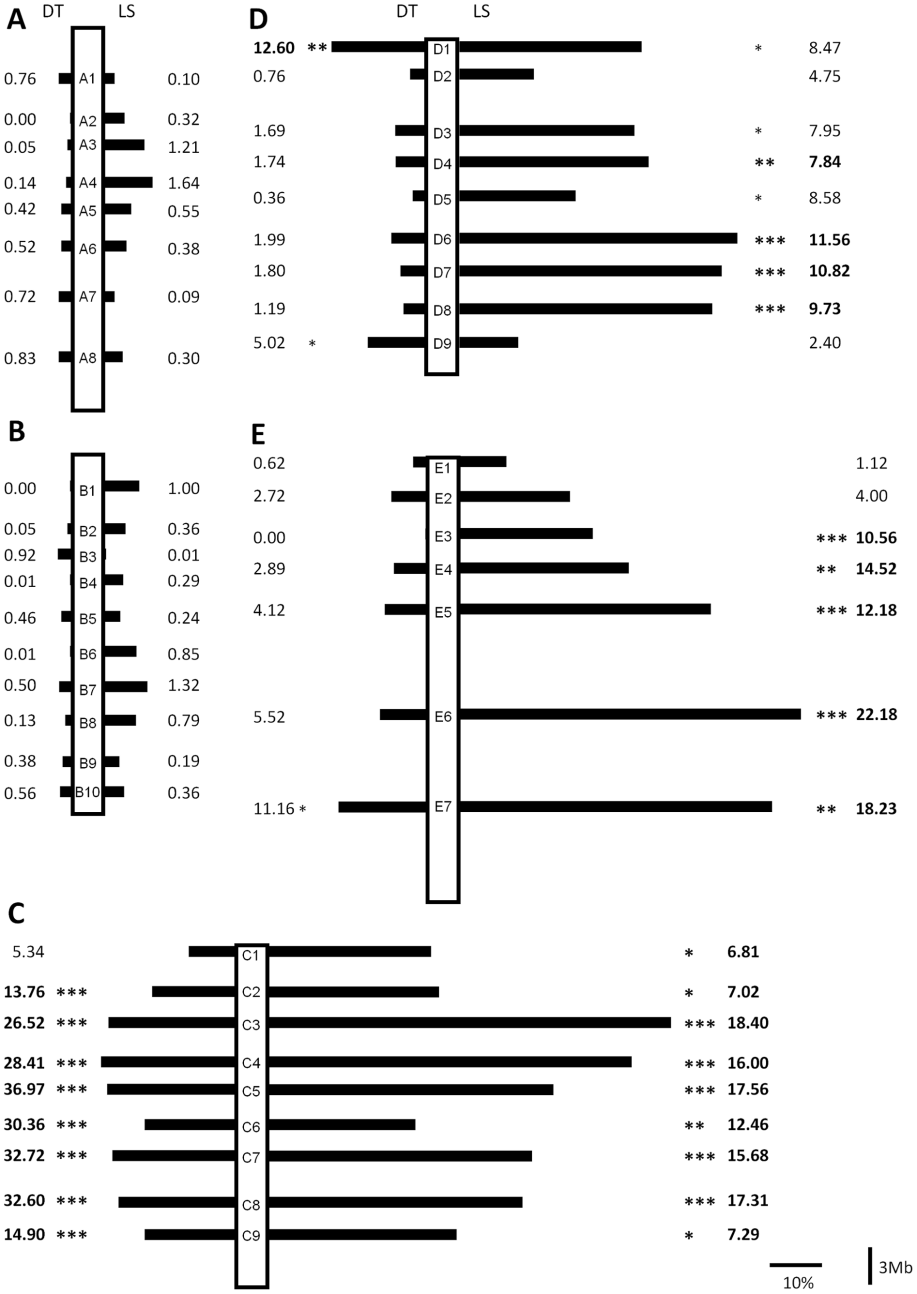
Schematic representation of the statistics computed for the F2 association studies involving the H5♂xW11♀ cross for developmental time (DT) and life span (LS). Black bars show the difference (in percentage) between genotypic classes that show the highest and lowest values for these two phenotypic traits ((highest value class – lowest value class)/lowest value class). The values next to the bars are the percentage of variation explained by each marker (the square of the Pearson correlation coefficient). For this calculation, we use the mean of each genotypic class as the expected value for that class. Significant associations are represented by *(0.05>P>0.01), **(0.01>P>0.001) and ***(P<0.001). Muller’s elements A – E are shown in A) to E), respectively.

**Figure 2:**
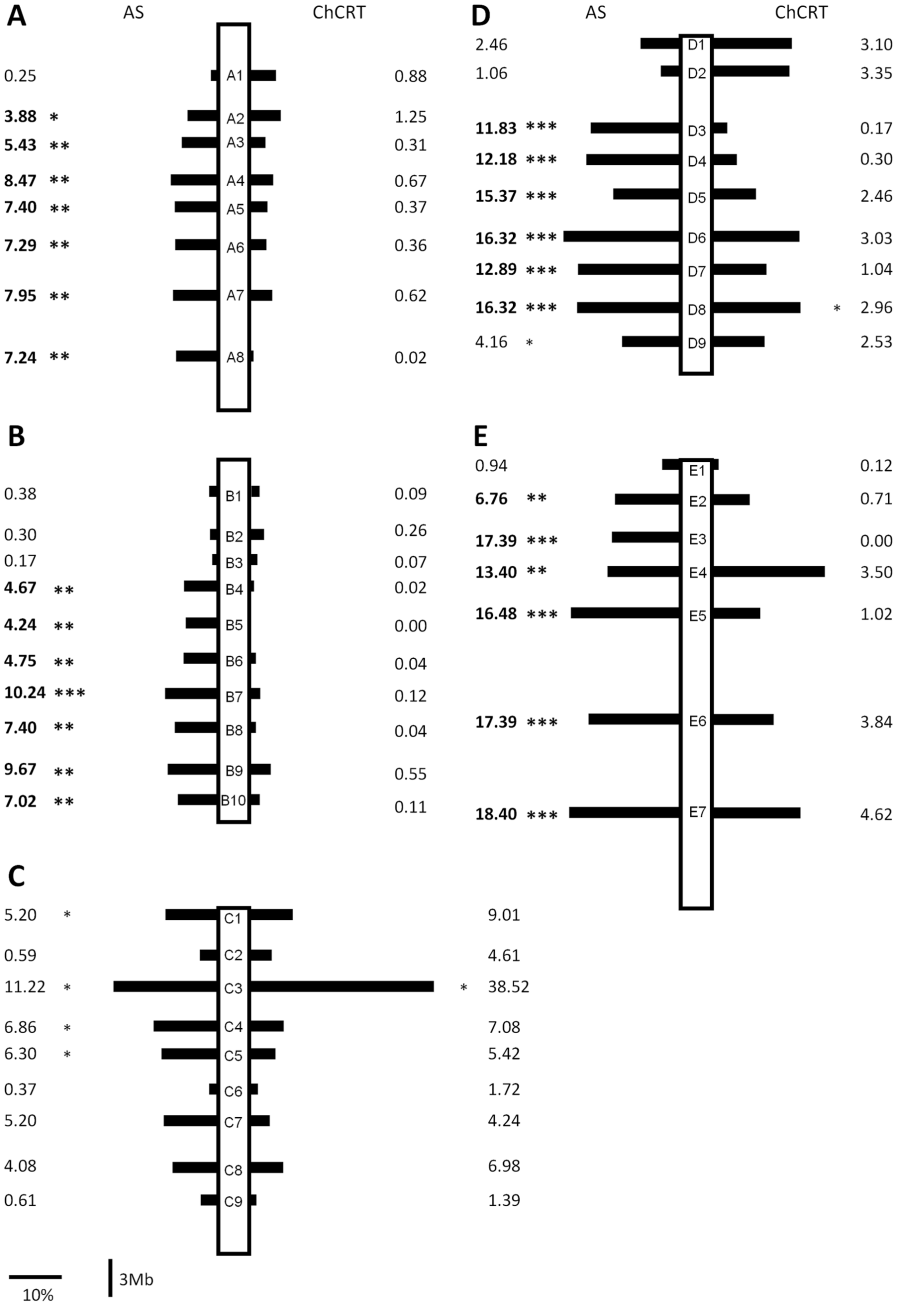
Schematic representation of the statistics computed for the F2 association studies involving the H5♂xW11♀ cross for abdominal size (AS) and chill-coma recovery time (CRT). Black bars show the difference (in percentage) between genotypic classes that show the highest and lowest values for these two phenotypic traits ((highest value class – lowest value class)/lowest value class). The values next to the bars are the percentage of variation explained by each marker (the square of the Pearson correlation coefficient). For this calculation, we use the mean of each genotypic class as the expected value for that class. Significant associations are represented by *(0.05>P>0.01), **(0.01>P>0.001) and ***(P<0.001). Muller’s elements A – E are shown in A) to E), respectively.

In the F2 association study involving strains H5 and W11, significant associations were found between the large indel markers here reported and DT, AS and LS (Figs. 1 and 2; Tables S2–S4). All associations between indel markers and CRT were non-significant after Bonferroni correction for multiple testing (Fig. 2; Table S5).

For DT, there are nine molecular markers showing significant associations after applying the sequential Bonferroni correction for multiple comparisons, one on Muller’s element D and eight on Muller’s element C (Fig. 1). The strongest signal comes from Muller’s element C where 64 out of 307 (20.8%) putative candidate genes fall into this region (Figs. 1 and 2; Table S6). The molecular markers showing significant associations explain as much as 36.97% of the total variation in DT found in this cross (see the C5 marker; Fig. 1 and Table S2; for this marker the difference between the genotypic classes showing extreme values for DT is 4.01 days (14.83 vs 18.84 days)). When using all markers showing a significant association with DT after Bonferroni correction, and stepwise regression, as much as 52.4% (molecular markers C4+C6+D1) of the total phenotypic variation in DT is explained.

In the case of LS, 18 associations remained significant after sequential Bonferroni correction, four on Muller’s element D, five on Muller’s element E and nine on Muller’s element C (Fig. 1). Twenty six, 18 and 26 putative candidate genes out of 139 (50.4%) are located in these chromosomal regions (Figs. 1 and 2; Table S6). The molecular markers showing significant associations explain as much as 22.18% of the total variation in LS found in this cross (see the E6 marker; Fig. 1 and Table S3; for this marker the difference between the genotypic classes showing extreme values for this trait is 30.18 days (41.89 vs 72.07 days)). When using all markers showing a significant association with LS after Bonferroni correction, and stepwise regression, as much as 50.3% (molecular markers D8+C7+E3) of the total phenotypic variation in LS is explained.

**Figure 3:**
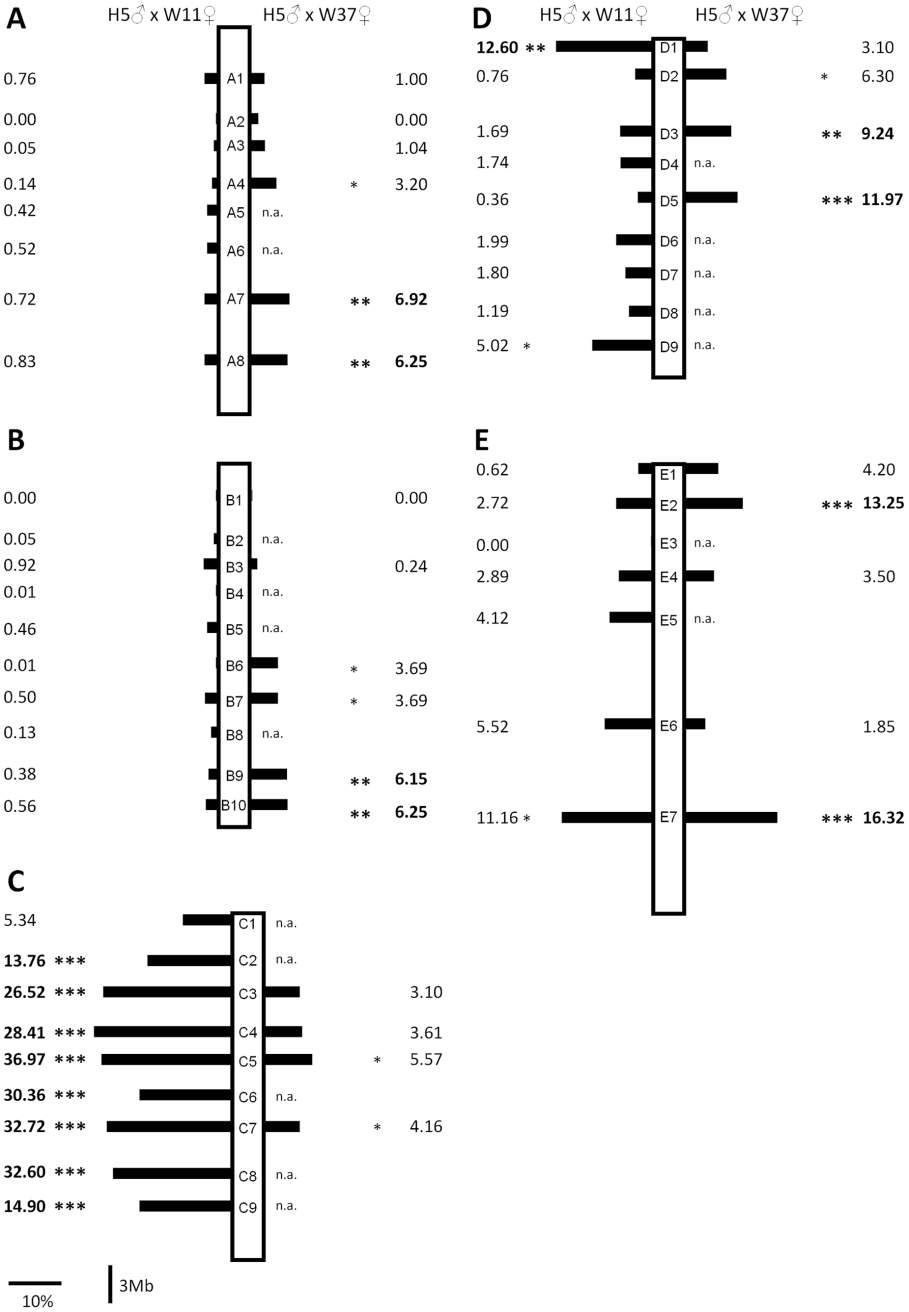
Schematic representation of the statistics computed for DT on the two F2 association studies (H5♂xW11♀ and H5♂xW37♀). Black bars show the difference (in percentage) between genotypic classes that developed faster and slower ((fast DT class – slow DT class)/slow DT class). The values next to the bars are the percentage of variation explained by each marker (the square of the Pearson correlation coefficient). For this calculation, we use the mean of each genotypic class as the expected value for that class. Significant associations are represented by *(0.05>P>0.01), **(0.01>P>0.001) and ***(p<0.001). n.a. – not applicable, the markers were not segregating in this F2 association study. Muller’s elements A – E are shown in A) to E), respectively.

Twenty six indel markers show significant associations with AS, namely six on each of the Muller’s element D and E and seven on each of the Muller’s elements A and B (Fig. 2). Thirty five putative candidate genes out of 40 (87.5%) are localized at the chromosomal regions showing significant associations (Figs. 1 and 2; Table S6). As much as 18.4% of the total variation in AS present in this cross is explained by a single molecular marker (see the E7 marker; Fig. 3 and Table S4; for this marker the difference between genotypic classes showing extreme values for AS is 0.20 relative units (0.85 vs 1.05 relative units where 1.00 represents the mean of the AS measured for all the phenotyped individuals). When all the markers showing a significant association with AS after Bonferroni correction are included in the stepwise regression analysis, as much as 33.4% (molecular markers E5+A4) of the total phenotypic variation in AS is explained.

It should be noted that some of the phenotypic traits analyzed here are significantly correlated (DT vs LS, Non-parametric Spearman’s correlation = −0.447; P<0.001 and AS vs CRT; Non-parametric Spearman’s correlation = −0.302; P<0.001). Correlations are also observed when the complete set of 975 phenotyped individuals is used rather than the 131 genotyped individuals (DT vs LS, Non-parametric Spearman’s correlation = −0.237; P<0.001; AS vs CRT; Non-parametric Spearman’s correlation = −0.160; P<0.001 and LS vs AS Non-parametric Spearman’s correlation = −0.176; P<0.001). This could be an indication that some genes account for variation in more than one of the traits analyzed here. Indeed, there are five genes that appear as putative candidates for more than one trait (Table 1). One of them (*EcR* located on Muller’s element C), is a putative candidate for variation in both DT and LS. The other four (*ilp2*, *Sk6*, *InR* and *Nf1*; the first two genes are located on Muller’s elements D and the other two on Muller’s element E) are putative candidates for both LS and AS. *EcR* (the only pleiotropic gene that according to our F2 association study could explain DT variation) is located at ∼0.8 Mbp from the indel marker C8 which alone explains as much as 32.6% of the total phenotypic variation for DT. The other four genes are putative pleiotropic candidates genes for AS, and the indel markers closer to these genes are D4 (∼0.4 and ∼1.4 Mbp from *ilp2* and *S6k* respectively) and E6 (∼1.5 and ∼2.4 Mbp from *Nf1* and *InR*, respectively). Using stepwise regression analysis, the marker on Muller’s element D is excluded from the model and the marker E6 explains, as much as 18.7% of the total variation observed for AS in the F2 association study. Finally, all five pleiotropic genes (*EcR, ilp2*, *Sk6*, *InR* and *Nf1*) were implicated in LS. Using stepwise regression analysis, the molecular marker D4 is excluded from the model, and the indel marker C8 along with E6 explain as much as 37.7% of the variation observed for LS.

**Table 1:** List of putative candidate genes that could account for variation in more than one phenotypic trait (pleiotropic genes).

Gene	Location (*D. virilis*)	In betweenmolecular markers	DT	LS	AS
*EcR* (C)	scaffold_12875∶4,666,484.4,709,770	C7–C8	0.000***	0.000***	
*ilp2* (C)	scaffold_13049∶8,664,534.8,665,005	D4–D5		0.007**	0.000***
*S6k* (D)	scaffold_13049∶9,771,911.9,782,583	D4–D5		0.007**	0.000***
*InR* (D)	scaffold_13047∶6,346,220.6,353,846	E5–E6		0.000***	0.000***
*Nf1* (E)	scaffold_13047∶5,385,397.5,395,947	E5–E6		0.000***	0.000***

The Muller’s element is shown within brackets after the gene name. Significant associations are denoted by **(0.001<P<0.01) and ***(P<0.001). It should be noted that the reported associations are with the markers (see Tables S2–S4) and not with the genes.

### H5♂xW37♀ F2 Association Study

In the H5♂xW11♀ F2 association study, a single marker, located close to the *Ecdysone receptor* (*EcR*) gene, explained as much as 32.6% of the total variation in DT. Therefore, in order to address whether large differences in DT are always due to variation in this genome region we performed a second F2 association study where the slow developing strain W11 was replaced by another slow developing strain (W37). This was feasible since 25 out of the 43 indel markers (58.1%) developed based on the H5 and W11 genomes were also segregating in the H5♂xW37♀ cross. Eight significant associations were found between the molecular markers and DT, namely two on each of the Muller’s elements A, B, D and E (Fig. 3). Markers on Muller’s element E explain as much as 16.3% of the total variation in DT found in this cross (see the marker E7; Fig. 3 and Table S7; for this marker the difference between the genotypic classes showing extreme values for DT is 2.79 days (14.46 vs 17.25 days)). When using all markers showing a significant association with DT after Bonferroni correction, and stepwise regression, as much as 40.5% (molecular markers E7+A7+E2) of the total phenotypic variation in DT is explained. Interestingly, none of these eight significant associations co-localized with those previously found in the other F2 association study.

## Discussion

In the H5♂xW11♀ F2 association study presented here, flies were phenotyped for four traits (DT, CRT, AS and LS) and associations were found between markers and all traits with the exception of CRT. It should be noted that significant correlations were found between DT and LS and AS and LS, which suggests the involvement of pleiotropic genes in the setting of these phenotypic traits. This possibility is further supported by the observation of significant associations between the same genome region and different traits. In *D. melanogaster*, DT has been found to be correlated with other life-history traits, such as adult weight at eclosion [18], adult size [18]–[21], pre-adult survival [19], [20] and longevity [22]. Moreover, a negative correlation between body size and longevity has been described for some mammals [23], [24], although for *Drosophila* the relationship between these two phenotypes is less obvious (see for instance [25]).

There are plenty of candidate genes reported to be associated with variability in DT, AS and LS in *D. melanogaster* (see for instance [26]–[28]). When using general GO terms, 169 *D. americana* genes could in principle explain the observed genotype-phenotype associations. Although these represent 34.8% of the total number of putative candidate genes, they are still too many to study in detail. Five genes (*EcR*, *ilp2*, *S6k*, *InR* and *Nf1*) can in principle account for variation in more than one trait. *InR*, *ilp2* and *S6k* are members of the insulin-signaling pathway that along with *Nf1* have been implicated in the determination of body size [28]–[30] and adult life span [31]–[34]. *EcR* has been shown to have a role in development during metamorphosis [35] and it is involved in the regulation of longevity [36]. These five genes might explain as much as 18.7, 32.6 and 37.7% of variation in AS, DT and LS, respectively in this particular cross. This is respectively 56.0%, 62.2% and 75.0% of the variation that is explained when using all markers that show a significant association with these traits after Bonferroni correction. Therefore, in *Drosophila*, genus-wide, a fraction of the variation in likely ecologically relevant traits (such as DT, AS and LS) might be due to pleiotropic genes.

In the H5♂xW11♀ F2 association study a significant association was obtained for almost the entire Muller’s element C. In *D. melanogaster*, Mensch et al. [37] found natural allelic variants that contribute to variation in DT for the genes *mastermind*; *invected*, *cricklet* and *CG14591*, that are all located on Muller’s element C. Therefore the result we get for *D. americana* could be taken as evidence for the presence of several genes influencing DT in this Muller’s element. Nevertheless, we cannot rule out the possibility of the involvement of a single gene of major effect associated with a low recombination rate along Muller’s element C as the explanation for our result. One such gene could be *EcR* that is located close to a marker that explains as much as 32.6% of the total variation in DT. In order to determine whether large differences in DT are always due to variation in this genome region we performed a second F2 association study where the slow developing strain W11 was replaced by another slow developing strain (W37). None of the statistically significant genotype-phenotype associations found in this second F2 association study co-localized with those obtained in the first one. Therefore, *EcR* cannot explain DT differences in the second study. One possibility is that the experimental design of the first study (selecting only individuals that show extreme phenotypes simultaneously for the four traits surveyed) favors the identification of this pleiotropic gene. Indeed, the correlation between DT and LS is higher when the set of 131 individuals showing extreme phenotypes (Non-parametric Spearman’s correlation = −0.447; P<0.001) is compared with the entire dataset (975 individuals; Non-parametric Spearman’s correlation = −0.237; P<0.001). Huang et al. [38] have reported that epistasis is an extremely important factor at defining variation for quantitative traits. Thus, another possible explanation is that the use of a different strain showing different alleles may have disrupted the interactions between *EcR* and other genes associated with developmental time differences. Other epistatic interactions could have been created in the second study resulting in different regions showing statistically significant associations. Therefore, at present, it seems likely that, in *D. americana*, variation in DT is due to the involvement of the pleiotropic gene *EcR*, but also of multiple other genes located across the entire genome. Compatible with this view, in *D. melanogaster*, about 65% of the 178 mutant lines analyzed by Mensch et al. [26] showed altered DT.

Despite the large number of candidate genes for DT, there is one region in the H5♂xW11♀ F2 association study that shows a significant association with this trait and for which there is no candidate gene (see marker D1; Fig. 3 and Table S6). This may be due to incomplete knowledge on genes that affect DT variation in *Drosophila*, but we cannot rule out the possibility that *D. americana* lineage specific genes are responsible for such an association. Such lineage-specific genes can only be identified using an approach that does not rely on candidate genes identified in other species.

It could be argued that the latitudinal and altitudinal gradients for DT that are found in *D. melanogaster* [39]–[41] and in *D. buzzatii* [42], and that are likely also present in *D. americana* (see [16]) could imply the presence of at least some common variants influencing DT in natural populations. Therefore, it is still conceivable that common variants will be found in *D. americana* when performing F2 association studies involving individuals from the extremes of the distribution. Nevertheless, performing such association studies may be difficult due to the suppression of recombination effect caused by the presence of the *X*/*4* fusion (Muller’s elements A/B) and six inversions (*Xc*, *2b*, *4ab*, *5a* and *5b;* on Muller’s elements A, E, B, C and C, respectively [43]), that are found at very different frequencies in populations from the extremes of the distribution.

Overall, our results illustrate not only the role of pleiotropic genes belonging to the insulin and ecdysone pathways, but also of other genes in the setting of likely ecologically relevant phenotypic traits. The set of 43 cheap and easy to type large indel markers distributed along the five major chromosomes of *D. americana* that was developed here is a tool that will ease the identification of the causative genes.

## Supporting Information

Figure S1 **Schematic representation of the indel markers developed for all five large** ***D. americana*** **chromosomal arms (Muller’s elements A – E are shown in A) to E), respectively).** The order is the same in every panel: from left to the right is shown the 1 Kb DNA ladder (GeneRuler™ 1 Kb DNA ladder from Thermo Scientific, USA), the H5, W11 and W37 strains. Some of the variants found in the H5 individuals involved in the H5♂ x W11♀ cross were not segregating in the H5♂ x W37♀ cross (markers B2, B5, C6, D8, E3) and therefore these molecular markers could not be used in the second study.
(TIF)

Table S1 **Primers used for the 43 equidistant molecular markers distributed along the** ***D. americana*** **genome based on the two completely sequenced** ***D. americana*** **strains (H5 and W11; Fig. S1).**
(PDF)

Table S2 **Results of the F2 H5♂xW11♀ association study for developmental time.**
(XLS)

Table S3 **Results of the F2 H5♂xW11♀ association study for life span.**
(XLS)

Table S4 **Results of the F2 H5♂xW11♀ association study for abdominal size.**
(XLS)

Table S5 **Results of the F2 H5♂xW11♀ association study for chill coma recovery time.**
(XLS)

Table S6 **List of candidate genes located near the chromosomal regions showing statistically significant associations after Bonferroni correction.**
(XLSX)

Table S7 **Results of the F2 H5♂xW37♀ association study for developmental time.**
(XLSX)
