# Supplementary figures and images for: Genes Belonging to the Insulin and Ecdysone Signaling Pathways Can Contribute to Developmental Time, Lifespan and Abdominal Size Variation in Drosophila americana
Source: PLoS One. 2014 Jan 28;9(1):e86690. doi: 10.1371/journal.pone.0086690 (PMC3904916; doi:10.1371/journal.pone.0086690)

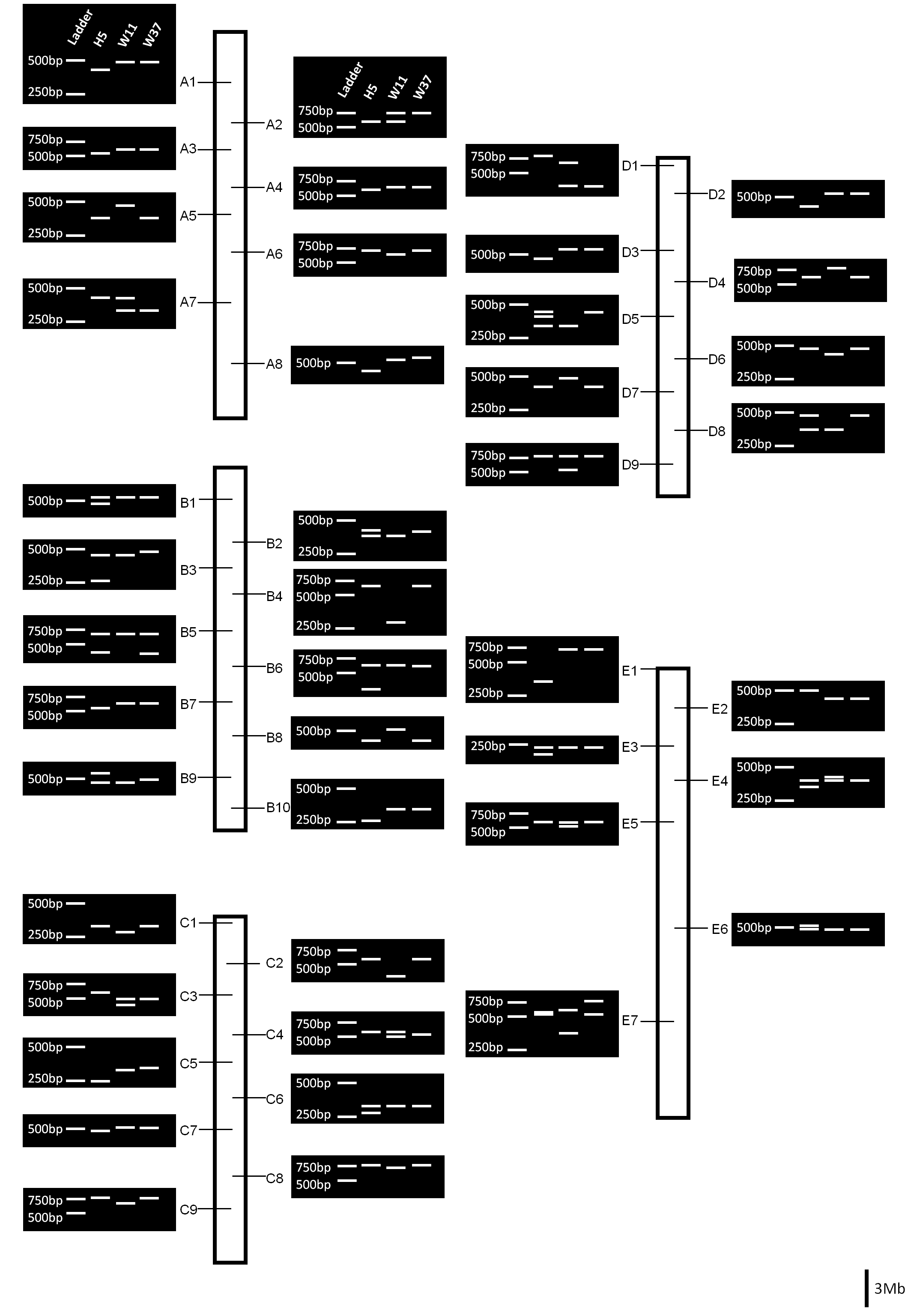

Supplement: Figure S1 — Schematic representation of the indel markers developed for all five large D. americana chromosomal arms (Muller’s elements A – E are shown in A) to E), respectively). The order is the same in every panel: from left to the right is shown the 1 Kb DNA ladder (GeneRuler™ 1 Kb DNA ladder from Thermo Scientific, USA), the H5, W11 and W37 strains. Some of the variants found in the H5 individuals involved in the H5♂ x W11♀ cross were not segregating in the H5♂ x W37♀ cross (markers B2, B5, C6, D8, E3) and therefore these molecular markers could not be used in the second study. (TIF) [file pone.0086690.s001.tif]
