# Supplementary material for: Genes Belonging to the Insulin and Ecdysone Signaling Pathways Can Contribute to Developmental Time, Lifespan and Abdominal Size Variation in Drosophila americana
Source: PLoS One. 2014 Jan 28;9(1):e86690. doi: 10.1371/journal.pone.0086690 (PMC3904916; doi:10.1371/journal.pone.0086690)
Supplement: Table S1 — Primers used for the 43 equidistant molecular markers distributed along the D. americana genome based on the two completely sequenced D. americana strains (H5 and W11; Fig. S1). (PDF) [file pone.0086690.s002.pdf]

Table S1. Primers used for the 43 equidistant molecular markers distributed along the entire *D. americana* genome based on the two completely sequenced *D. americana* strains (H5 and W11; Fig. 1).

Markers (Muller's Element/number)	Primer	Sequence	Primer	Sequence	Annealing temperature
A1	W11_1089882_F	GAGCATTTCGCACAGATT	W11_1089882_R	TCGTTCGCATTGGGTTTA	51
A2	W11_1087475_F	CTTTGTGGTTGGTCTCAG	W11_1087475_R	AGCAGCAGTAGTTGTAGG	48
A3	W11_N1169_F	ATAAATGGCAAAACAGTG	W11_N1169_R	TAAACGATACGAGAACCT	48
A4	W11_1062908_F	TCCTCAAGCCTACACAAA	W11_1062908_R	CCAACATTTTCATTCATC	48
A5	W11_1090885_F	CGCCGATTTCATTTTCAT	W11_1090885_R	GCACGCACTCAGACTTTT	48
A6	H5_1262043_F	AGTGAGGGAGAGCATAGG	H5_1262043_R	GCATTGTTTGTTTGAAGA	48
A7	H5_1283957_F	GCCTTGCCGTCTTTCCAT	H5_1283957_R	GCCACTTGCCACATTTTG	53
A8	W11_1085512_F	TTTTGGCGGATTCTGCTG	W11_1085512_R	TATGCGTGGCTGTCGTCT	50
B1	W11_1078199_F	ATTTGTGTTGGGGGTAAG	W11_1078199_R	TCTGGTCTGGTCTGTTCT	50
B2	H5_N188373_F	CTGTTTGGTTAGGTTTCC	H5_N188373_R	TGCGTTGGTTGATTTTTC	49
B3	W11_1076541_F	CTGATACCCTACGCTTGG	W11_1076541_R	GTTGTCGCTGCTGCTGTT	49
B4	H5_N96096_F	CACATTTTACGCTTCTTC	H5_N96096_R	ATTCTTTCAACCATTATT	48
B5	W11_N1824_F	CGGCTTCTTGATTGACAT	W11_N1824_R	GCATCTTGGTGGTGGTAA	49
B6	W11_1081739_F	GCTAACAACTGCTCTACG	W11_1081739_R	CTACTCTCTGCGGACTCG	48
B7	W11_1088703_H5F	AATGTGCTTTGCGAGTCA	W11_1088703_R	GCTGTTGGTCTGTGTTCA	48
B8	W11_1095820_F	ACACAAGGCGAACATTTA	W11_1095820_R	TTGACCGATTTGCTTTAC	49
B9	H5_sc5421_F	CGTTAGAGCCAGAAAGAC	H5_sc5421_R	AGATGAAGAGGAGGATAC	48
B10	W11_N7121_F	ACGAGTCCTGTGTCAAAT	W11_N7121_R	TTTTTATTGCTTTTTCTT	48
C1	H5_sc13321_F	CGAAATAAGGTCAACAAG	H5_sc13321_R	AATAAGTCTGCGAATAGC	50
C2	H5_1283021_F	TTAGACAAACAGCAAAGG	H5_1283021_R	TTCAAAAGAGCCACCATA	50
C3	H5_sc801_F	CTGGGAGATGTTGAAGTA	H5_sc801_R	GTGTTTGGAGAGGTGTCG	48

C4	H5_1273029_F	GCCAAAGACAATACAAAG	H5_1273029_R	AGAAAAGAGACGACATCC	48
C5	W11_1086383_F	CAAGACTAATCCCTAASG	W11_1086383_R	CCAAAACAACAAGACTCA	48
C6	W11_1110251_F	GTCGGGAACCTCGTGAAC	W11_1110251_R	TAAGAATCGGCGTTGGAA	52
C7	H5_sc48421_F	CAGCAGGAAATGGATAGG	H5_sc48421_R	ATTGGGCGGGTAACTCTT	49
C8	H5_1259877_F	ATGCTGATGGGCTCTGAT	H5_1259877_R	ATTCGTTGCTTTCTGTGC	51
C9	H5_1266174_F	GTGGACGCTCGCTAAATC	H5_1266174_R	GTCGCTGGCTTTGTGTGC	51
D1	H5_N97693_F	GGTAATGGGTAAAAATGC	H5_N97693_R	TCAAAATAACTGGGGTAA	48
D2	W11_sc69401_F	ACCAAAGATAAACTGACA	W11_sc69401_R	CTTACGGATTACCAACTT	48
D3	W11_1097237_F	TGAACTGAAAGGGTATGG	W11_1097237_R	TTGATGATGAATGAATGT	48
D4	W11_sc17011_F	TAGAAGGTGGCAGTGTTG	W11_sc17011_R	GCTGTGGACTCTGGAATA	50
D5	H5D_914_F	TTTTTCTGTTTTTCAATA	H5D_914_R	AATCAAATGCGTAGTAGG	46
D6	H5D_1093_F	AATGAGAGGGTGGCAAGC	H5D_1093_R	GCGAWAAAATGGGGAATA	50
D7	W11D_3223_F	TACGCCCCAAATAATGAC	W11D_3223_F	TGCCCAAAACAATGACCA	50
D8	H5D_1359_F	GCTTTTATCTTTTACCTT	H5D_1359_R	TCGTTGTTGACTTATGAG	48
D9	H5_sc14941_F	CATTTCCTTTAGCCATTC	H5_sc14941_R	CCATTCGTATTCAACATT	49
E1	W11_N9270_F	AACCAATCTGCCTGAACT	W11_N9270_R	CACAAACTCCCATAAAGC	50
E2	H5E_242_F	TCGCTGAGAAATAAAATG	H5E_242_R	AATACTAAACACCCTACC	48
E3	W11_N10570_F	AAAGTGAGGGAAGAGCAA	W11_N10570_R	CCGATTTAGGTTGAAGTG	50
E4	W11E_915_F	GCCTGATTTATTGACATT	W11E_915_R	ACGCACTTCCTGTATGAT	48
E5	H5E_625_F	TTTTCGGCTCTTGACATA	H5E_625_R	ATTTCTACCTGGGACACA	49
E6	H5E_1151_F	CTGAGACCTGGGAGAGAC	H5E_1151_R	TGTGAAAGTGGATAATGC	48
E7	Fst_PEST_F	CAGAAGAGCCCGTAGAAA	Fst_PEST_R	GGTGCCTTGGTAGTCTCG	56
